# Supplementary material for: Molecular tuning of sea anemone stinging
Source: bioRxiv. 2023 Aug 3:2023.06.15.545144. Preprint. [Version 2] doi: 10.1101/2023.06.15.545144 (PMC10418081; doi:10.1101/2023.06.15.545144)
Supplement: Supplementary file 1 [file NIHPP2023.06.15.545144V2-supplement-1.pdf]

bioRxiv preprint doi: https://doi.org/10.1101/2023.06.15.545144; this version posted August 3, 2023. The copyright holder for this preprint (which was not certified by peer review) is the author/funder, who has granted bioRxiv a license to display the preprint in perpetuity. It is made available under aCC-BY 4.0 International license.

Supplementary Information Text: Markov Decision Processes modeling defensive vs predatory stinging.

bioRxiv preprint doi: https://doi.org/10.1101/2023.06.15.545144; this version posted August 3, 2023. The copyright holder for this preprint (which was not certified by peer review) is the author/funder, who has granted bioRxiv a license to display the preprint in perpetuity. It is made available under aCC-BY 4.0 International license.

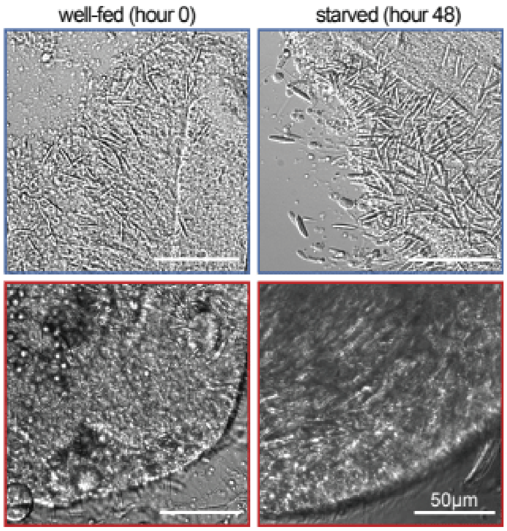

Figure 2S1. Modulation of *Nematostella* and *Exaiptasia* stinging is not due to changes in the abundance of nematocytes.

Nematocytes were highly abundant in tentacles from *Nematostella (top)* and *Exaiptasia (bottom)* before and after starvation. Representative of n = 3 animals. Scale bar = 50μm.

bioRxiv preprint doi: https://doi.org/10.1101/2023.06.15.545144; this version posted August 3, 2023. The copyright holder for this preprint (which was not certified by peer review) is the author/funder, who has granted bioRxiv a license to display the preprint in perpetuity. It is made available under aCC-BY 4.0 International license.

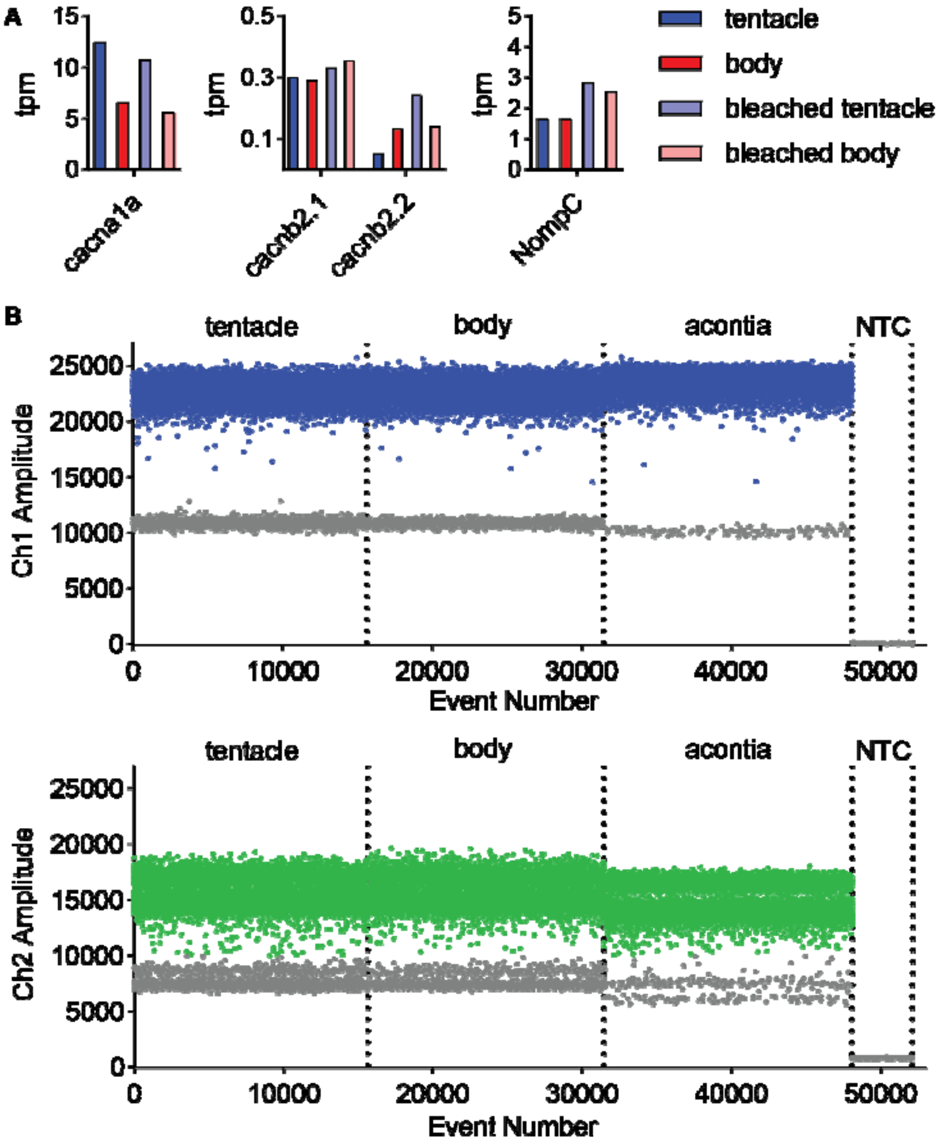

Figure 4S1. Transcriptomic and molecular analyses of *Exaiptasia* β subunit isoforms.

A) mRNA expression (transcripts per million, TPM) of voltage-gated calcium (Ca_V) channel α and β subunits in *Exaiptasia* tentacle (nematocyte abundant, blue), body (nematocyte non-abundant, red), bleached (minimal symbionts) tentacle (light blue), bleached body (light red) tissues. The Ca_V α subunit was identified by homology to the sequence of the cnidarian Ca_V2.1 homolog found enriched in *Nematostella* nematocyte-rich tissues (Weir et al., 2020). NompC, the putative mechanoreceptor in *Nematostella* nematocytes (Schüler et al., 2015; Weir et al., 2020), was also detected in *Exaiptasia* tentacles.

B) Representative plots of fluorescent amplitude across event number (droplet events) from amplification of unique regions of EdCa_Vβ1 (Ch1, *Top*) and EdCa_Vβ2 (Ch2, *Bottom*) sequences using droplet digital PCR (ddPCR, Bio-Rad Laboratories). Individual lanes correspond to tentacle RNA, body RNA, acontia RNA, and no template control (NTC). Blue and green points indicate positive PCR droplets after thresholding and gray points indicate negative droplets.

bioRxiv preprint doi: https://doi.org/10.1101/2023.06.15.545144; this version posted August 3, 2023. The copyright holder for this preprint (which was not certified by peer review) is the author/funder, who has granted bioRxiv a license to display the preprint in perpetuity. It is made available under aCC-BY 4.0 International license.

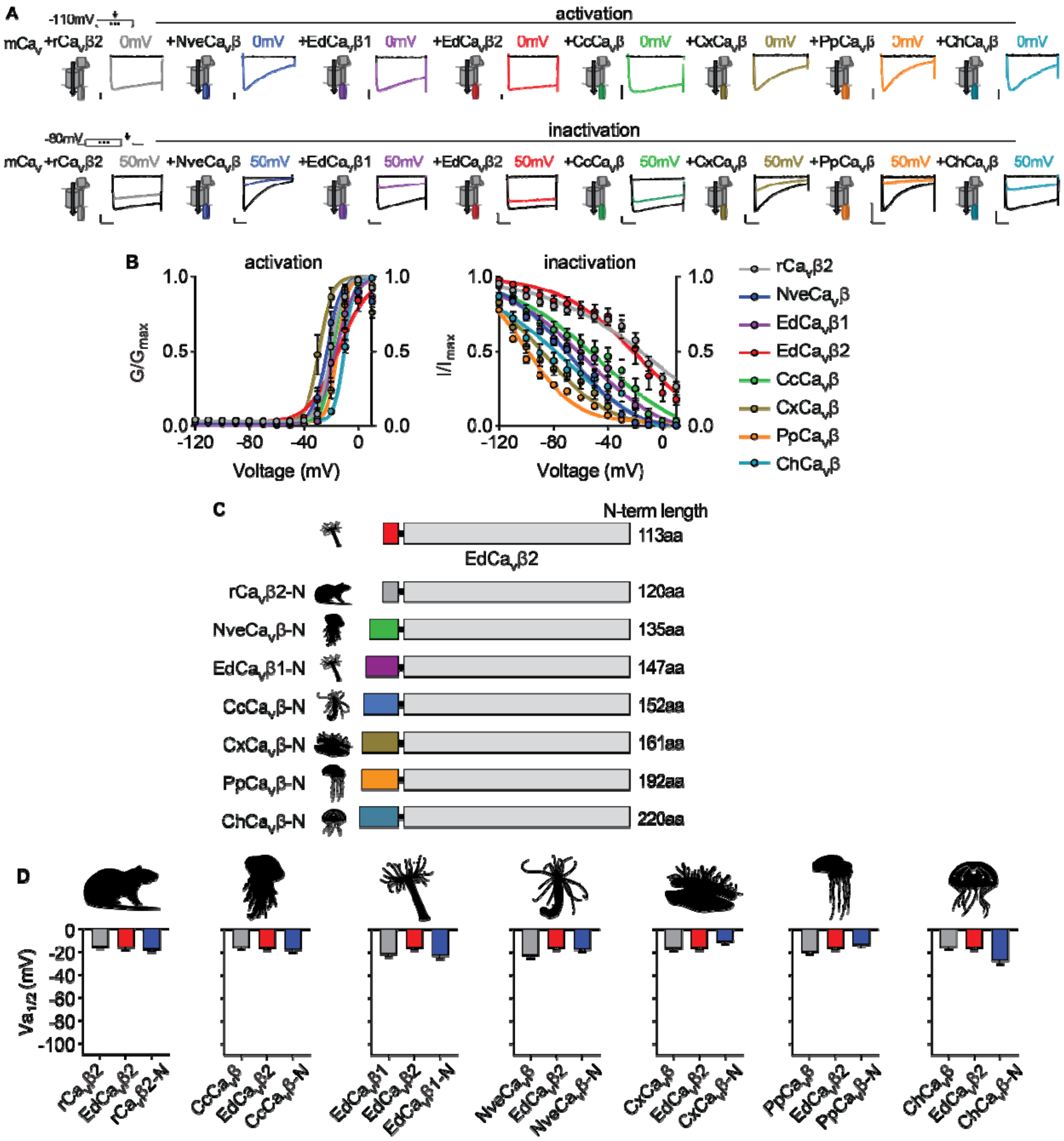

Figure 5S1. Voltage-dependent activation of Ca_V channels is conserved across cnidarian β subunits.

A) *Top*: Voltage-gated currents from heterologously-expressed chimeric Ca_Vs with the indicated β subunits elicited by voltage pulses to -120mV (no current, black) and 0mV (colored). Abbreviations of species: Nve, *Nematostella vectensis*; Ed, *Exaiptasia diaphana*; Cc, *Cyanea capillata* (jellyfish); Pp, *Physalia physalis* (siphonophore); Ch, *Clytia hemisphaerica* (jellyfish); Cx, *Cassiopea xamachana* (jellyfish); r, *Rattus norvegicus*. *Bottom*: Voltage-gated currents elicited by a maximally activating voltage pulse following 1 s pre-pulses to −110 mV (max current, black), −50 mV (colored), or 20 mV (inactivated, no current, black). Scalebars = 100pA, 50ms.

B) Activation and inactivation curves for heterologously-expressed chimeric Ca_Vs with different β subunits. Activation: $rCa_V\beta2$ $V_{a1/2}$ = -19.76 ± 1.16mV, n = 12; $NveCa_V\beta$ $V_{a1/2}$ = -23.07 ± 1.16mV, n = 5; $EdCa_V\beta1$ $V_{a1/2}$ = -18.27 ± 1.08mV, n = 8; $EdCa_V\beta2$ $V_{a1/2}$ = -14.22 ± 1.46mV, n = 5; $CcCa_V\beta$ $V_{a1/2}$ = -18.47 ± 1.59mV, n = 6; $CxCa_V\beta$ $V_{a1/2}$ = -28.89 ± 1.54mV, n = 15; $PpCa_V\beta$ $V_{a1/2}$ = -15.29 ±

bioRxiv preprint doi: https://doi.org/10.1101/2023.06.15.545144; this version posted August 3, 2023. The copyright holder for this preprint (which was not certified by peer review) is the author/funder, who has granted bioRxiv a license to display the preprint in perpetuity. It is made available under aCC-BY 4.0 International license.

1.23mV, n = 10; ChCa$_V$β $V_{a1/2}$ = -10.30 ± 1.04mV, n = 12. rCa$_V$β2 $V_{i1/2}$ = -2.98 ± 13.51mV, n = 12; NveCa$_V$β $V_{i1/2}$ = -68.93 ± 1.53mV, n = 5; EdCa$_V$β1 $V_{i1/2}$ = -56.76 ± 3.18mV, n = 8; EdCa$_V$β2 $V_{i1/2}$ = -18.84 ± 8.00mV, n = 5; CcCa$_V$β subunit $V_{i1/2}$ = -47.81 ± 5.57mV, n = 6; CxCa$_V$β $V_{i1/2}$ = -87.75 ± 1.72mV, n = 15; PpCa$_V$β $V_{i1/2}$ = -99.80 ± 0.92mV, n = 10; ChCa$_V$β $V_{i1/2}$ = -70.25 ± 4.67mV, n = 12.

C) Diagram of Ca$_V$ β subunit domain swaps and the length of the N-terminus swapped in amino acids.

D) Cnidarian Ca$_V$ β N-termini do not greatly affect voltage-dependent activation of Ca$_V$ channels containing EdCa$_V$β2. Voltage-dependent activation ($V_{a1/2}$) of heterologously-expressed Ca$_V$s with WT EdCa$_V$β2, β subunits from the indicated cnidarians, and chimeras with their N-termini on EdCa$_V$β2, $p = 0.5830$ for average $V_{i1/2}$ values across mutant beta subunits, one-way ANOVA with Bartlett’s test and post-hoc Tukey test, n = 4-7 cells. Data represented as mean ± sem.

bioRxiv preprint doi: https://doi.org/10.1101/2023.06.15.545144; this version posted August 3, 2023. The copyright holder for this preprint (which was not certified by peer review) is the author/funder, who has granted bioRxiv a license to display the preprint in perpetuity. It is made available under aCC-BY 4.0 International license.

Figure 5 Supplementary Table 1: Wild type and Chimeric $Ca_V\beta$ amino acid sequences.
